# Supplementary material for: Atmospheric dryness reduces photosynthesis along a large range of soil water deficits
Source: Nat Commun. 2022 Feb 21;13:989. doi: 10.1038/s41467-022-28652-7 (PMC8861027; doi:10.1038/s41467-022-28652-7)
Supplement: Supplementary file 1 — Supplementary Information [file 41467_2022_28652_MOESM1_ESM.pdf]

Supplementary Information

Atmospheric dryness reduces photosynthesis along a large range of soil water deficits

Zheng Fu[1*], Philippe Ciais[1], I. Colin Prentice[2, 3, 4], Pierre Gentine[5], David Makowski[6], Ana Bastos[7], Xiangzhong Luo[8], Julia K. Green[1], Paul C. Stoy[9], Hui Yang[1], Tomohiro Hajima[10]

[1] Laboratoire des Sciences du Climat et de l'Environnement, LSCE/IPSL, CEA-CNRS-UVSQ, Université Paris-Saclay, Gif-sur-Yvette, 91191, France

[2] Georgina Mace Centre for the Living Planet, Department of Life Sciences, Imperial College London, Silwood Park Campus, Buckhurst Road, Ascot, SL5 7PY, UK

[3] Department of Biological Sciences, Macquarie University, North Ryde, NSW 2109, Australia

[4] Ministry of Education Key Laboratory for Earth System Modeling, Department of Earth System Science, Tsinghua University, Beijing 100084, China

[5] Department of Earth and Environmental Engineering, Columbia University, New York, NY, 10027, USA

[6] Unit Applied mathematics and computer science (UMR 518) INRAE AgroParisTech Université Paris-Saclay, Paris, France

[7] Department Biogeochemical Integration, Max Planck Institute for Biogeochemistry, D-07745 Jena, Germany

[8] Department of Geography, National University of Singapore, Singapore

[9] Department of Biological Systems Engineering, University of Wisconsin – Madison, USA

[10] Research Center for Environmental Modeling and Application, Japan Agency for Marine-Earth Science and Technology, 3173-25 Showamachi, Kanazawaku, Yokohama, 236-0001, Japan

[*]Correspondence to: zheng.fu@lsce.ipsl.fr

Contents of this file

Supplementary Figures 1 to 16

Supplementary Tables 1 to 3

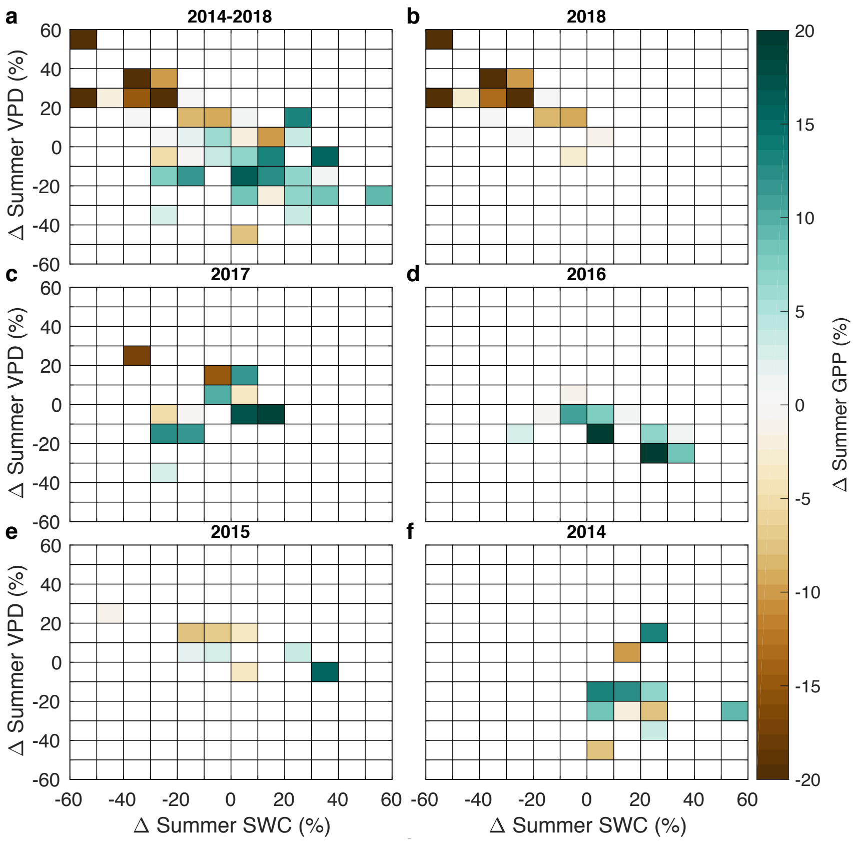

Supplementary Fig. 1 Response of the relative changes of summer gross primary production (GPP) to the relative changes of summer soil water content (SWC) and vapor pressure deficit (VPD) across European ecosystems during 2014–2018 (see Supplementary Table 1). **a**, Response of the relative changes of summer GPP to the relative changes of summer SWC and VPD during 2014–2018. **b-f**, Response of the relative changes of summer GPP to the relative changes of summer SWC and VPD in 2018 (**b**), 2017 (**c**), 2016 (**d**), 2015 (**e**) and 2014 (**f**).

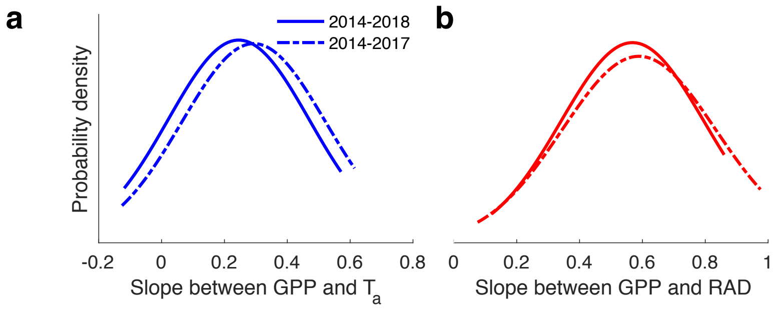

Supplementary Fig. 2 Probability distributions across all sites for the linear regression slope of daily gross primary production (GPP) anomalies to air temperature (T_a) and incoming shortwave radiation anomalies (RAD) during the summer across 2014–2018 and 2014–2017. **a-b**, Probability distributions across all sites for the linear regression slope of daily GPP anomalies to T_a (**a**) and RAD (**b**) during the summer across 2014–2018 and 2014–2017.

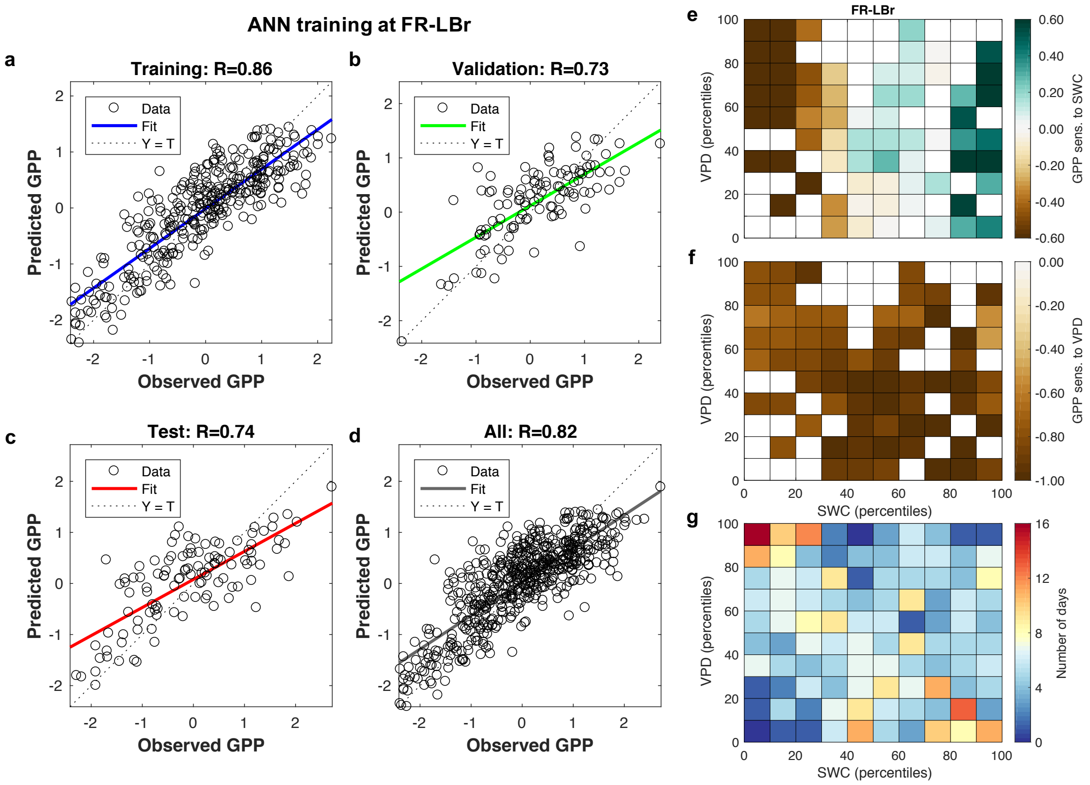

Supplementary Fig. 3 An example to show the sensitivity of gross primary production (GPP) to soil water content (SWC) and vapor pressure deficit (VPD) at FR-LBr (Supplementary Table 2). **a-d**, The regression plot of predicted GPP from Artificial Neural Network (ANN) and observed GPP for training (60% of data), validation (20% of data), testing (20% of data) and all data (100%). **e-f**, The sensitivity of GPP to SWC (**e**) and VPD (**f**). Bins with less than 5 data points are not used (white squares) to ensure reliability of results. **g**, The number of days at each bin. Daily temperature, VPD, SWC and incoming shortwave radiation were used as predictor variables while daily GPP is used as response variable. We limited the ANN fit to the small number of predictors that are known environmental drivers, in order to avoid over-fitting (see Methods).

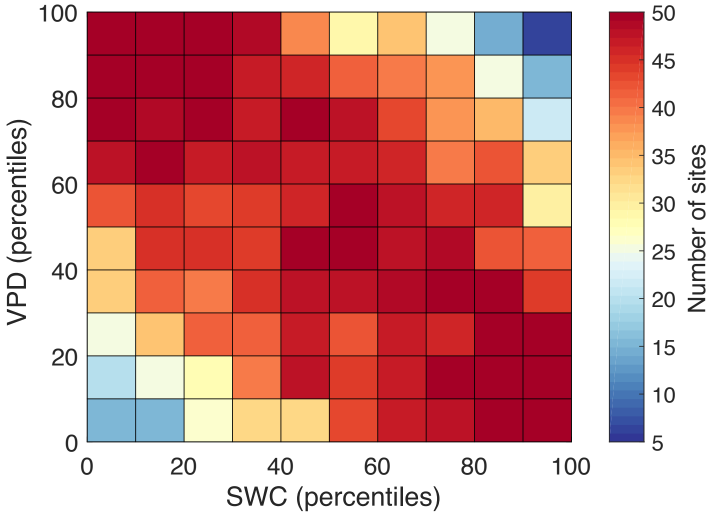

Supplementary Fig. 4 Distribution of the number of sites in each bin using ICOS and FLUXNET2015 data.

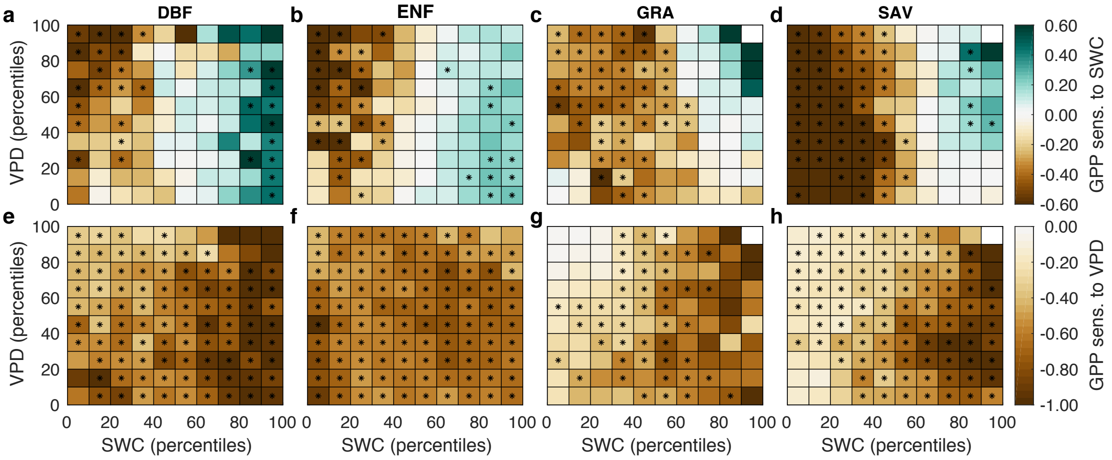

Supplementary Fig. 5 The sensitivity of gross primary production (GPP) to soil water content (SWC) and vapor pressure deficit (VPD) across different plant function types. **a-d**, The sensitivity of GPP to SWC across different plant function types. **e-h**, The sensitivity of GPP to VPD across different plant function types. DBF: broadleaved deciduous forests; ENF: evergreen needle-leaved forests; GRA: grasslands; SAV: savannas. Mixed forests, evergreen broadleaved forests and shrublands are not shown due to the limitation of small number sites. '*' represents the sensitivities are significantly different from zero by t-tests (p<0.05) across all sites.

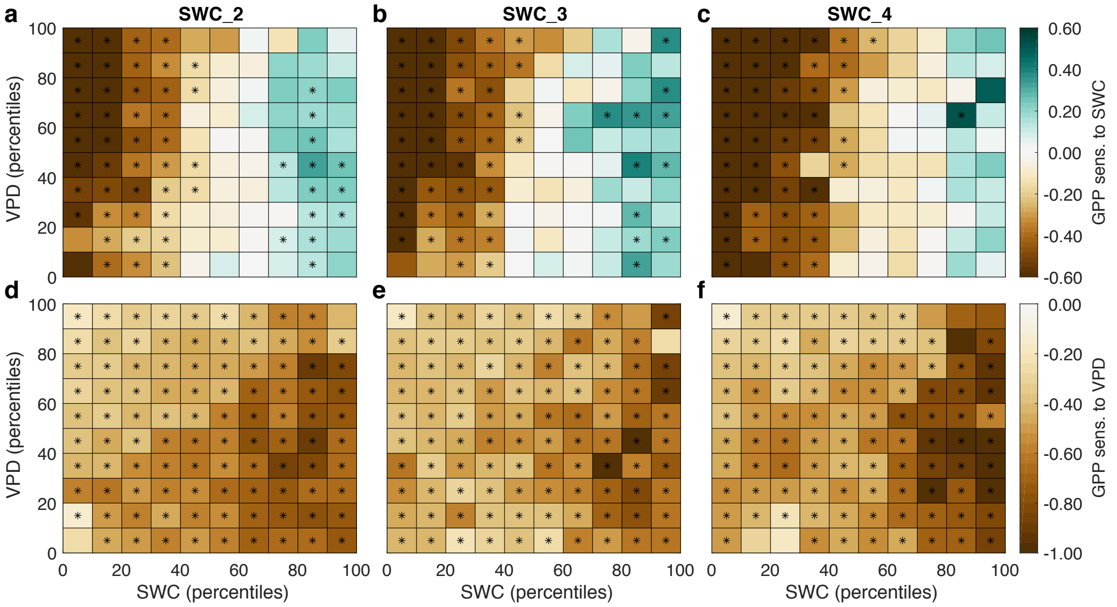

Supplementary Fig. 6 The sensitivity of gross primary production (GPP) to soil water content (SWC) and vapor pressure deficit (VPD) using the SWC in the second (SWC_2), third (SWC_3), and fourth (SWC_4) depths, respectively (2-4: increases with the depth, 4 is deepest). **a-c**, The sensitivity of GPP to SWC using the SWC in different soil depths. **d-f**, The sensitivity of GPP to VPD using the SWC in different soil depths.

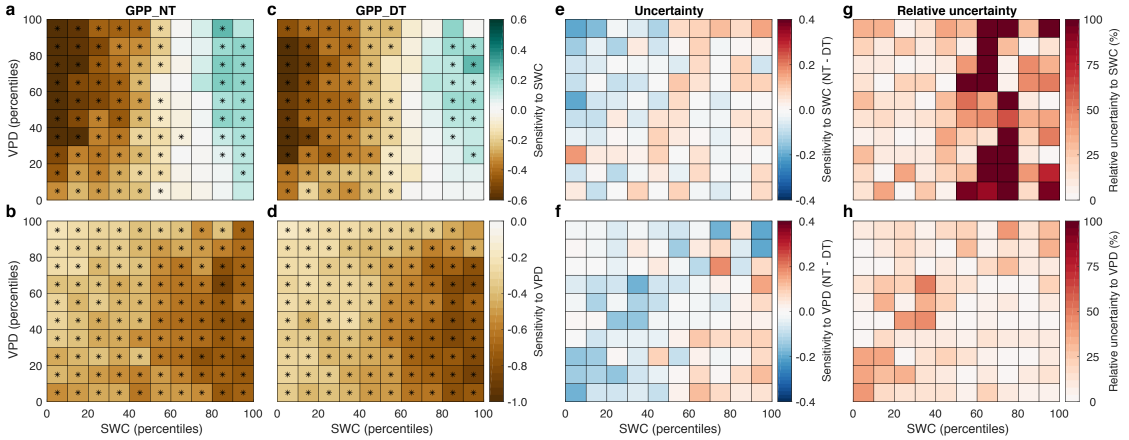

Supplementary Fig. 7 Effect of the partitioning methods (Nighttime vs. Daytime) on gross primary production (GPP) sensitivity to soil water content (SWC) and vapor pressure deficit (VPD). The sensitivity of GPP to SWC (**a**) and VPD (**b**) using GPP from nighttime partitioning method (GPP_NT). The sensitivity of GPP to SWC (**c**) and VPD (**d**) using GPP from daytime partitioning method (GPP_DT). Uncertainty in GPP sensitivity to SWC (**e**) and VPD (**f**). Relative uncertainty in GPP sensitivity to SWC (**g**) and VPD (**h**). Please note that the high levels of relative uncertainty occurred in the bins with statistically insignificant sensitivity values (**a**, **c**, **g**). Since these sensitivity values are close to zero, a low absolute uncertainty leads to a high relative uncertainty.

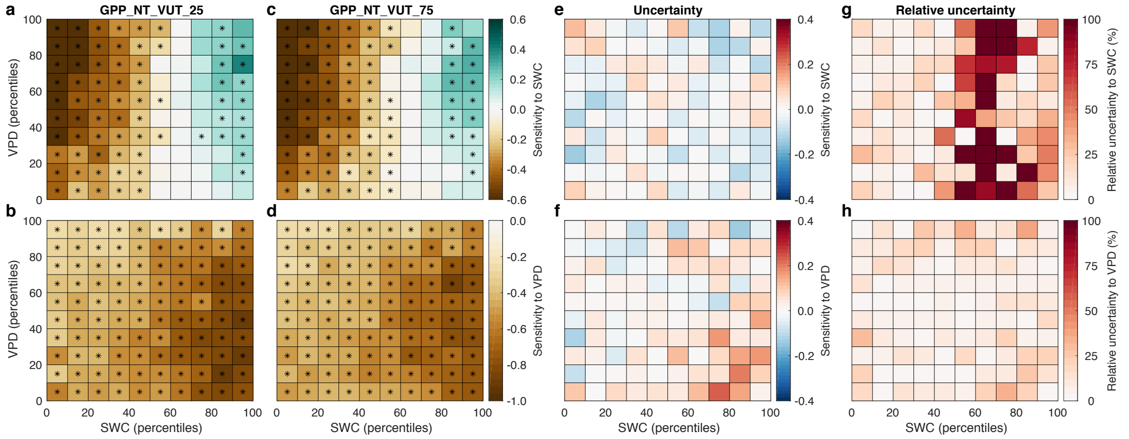

Supplementary Fig. 8 Effect of the net ecosystem CO_2 exchange processing on gross primary production (GPP) sensitivity to soil water content (SWC) and vapor pressure deficit (VPD). The sensitivity of GPP to SWC (**a**) and VPD (**b**) using GPP_NT_VUT_25. The sensitivity of GPP to SWC (**c**) and VPD (**d**) using GPP_NT_VUT_75. Uncertainty in GPP sensitivity to SWC (**e**) and VPD (**f**). Relative uncertainty in GPP sensitivity to SWC (**g**) and VPD (**h**). Please note that the high levels of relative uncertainty occurred in the bins with statistically insignificant sensitivity values (**a**, **c**, **g**). Since these sensitivity values are close to zero, a low absolute uncertainty leads to a high relative uncertainty.

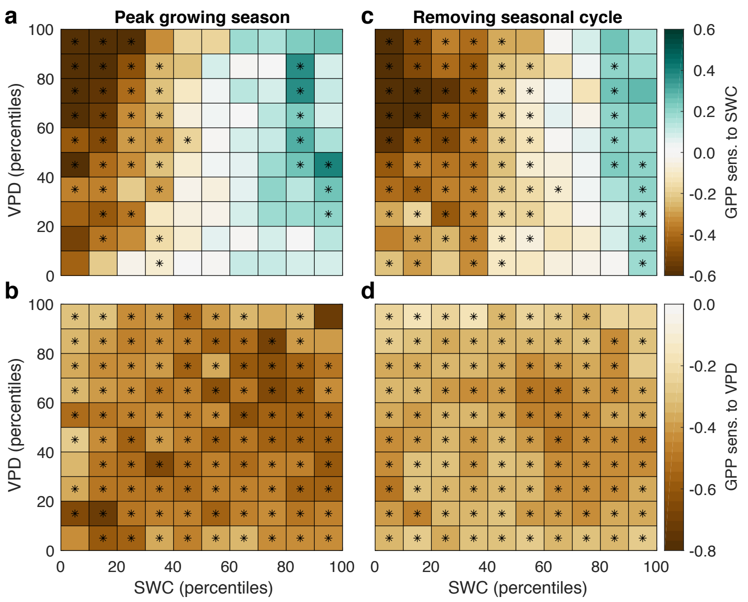

Supplementary Fig. 9 Effect of the phenological cycle on gross primary production (GPP) sensitivity to soil water content (SWC) and vapor pressure deficit (VPD). **a-b**, The sensitivity of GPP to SWC and VPD using only peak growing season, the 3-month period with the maximum mean GPP across the available years, where seasonal variability is muted. **c-d**, The sensitivity of GPP to SWC and VPD using anomalies by removing the seasonal cycle.

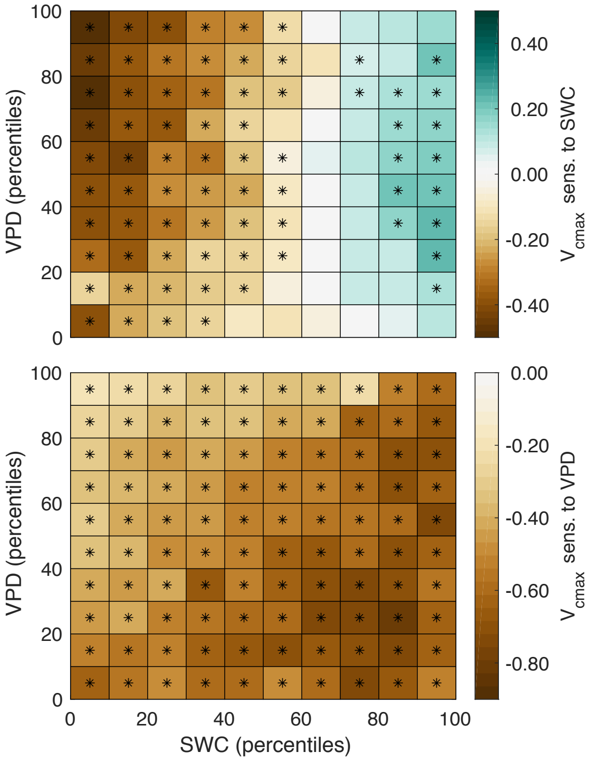

Supplementary Fig. 10 The sensitivity of maximum carboxylation rate (V_{cmax}) to soil water content (SWC) and vapor pressure deficit (VPD) using ICOS and FLUXNET2015 data. '*' represents the sensitivities are significantly different from zero by t-tests (p<0.05) across all sites.

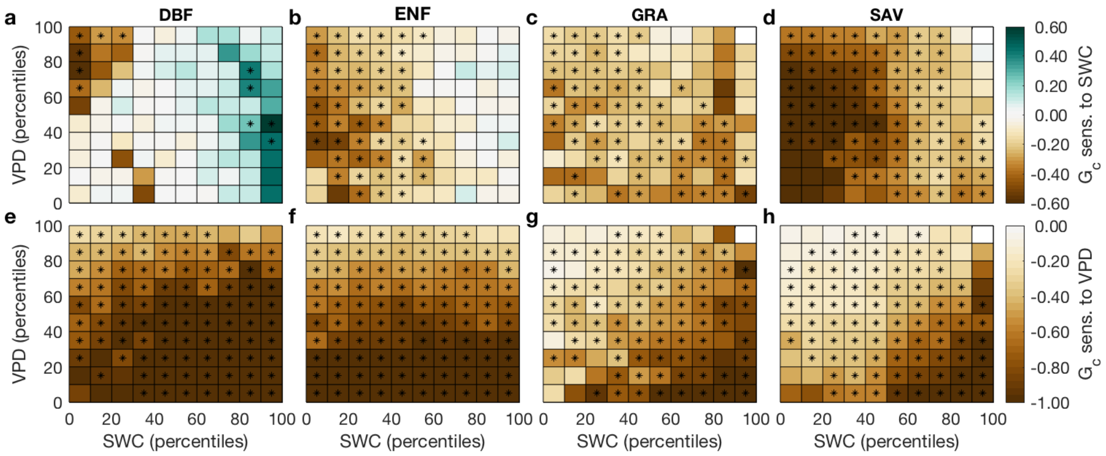

Supplementary Fig. 11 The sensitivity of canopy conductance (G_c) to soil water content (SWC) and vapor pressure deficit (VPD) across different plant function types. **a-d**, The sensitivity of G_c to SWC across different plant function types. **e-h**, The sensitivity of G_c to VPD across different plant function types. DBF: broadleaved deciduous forests; ENF: evergreen needle-leaved forests; GRA: grasslands; SAV: savannas. '*' represents the sensitivities are significantly different from zero by t-tests (p<0.05) across all sites.

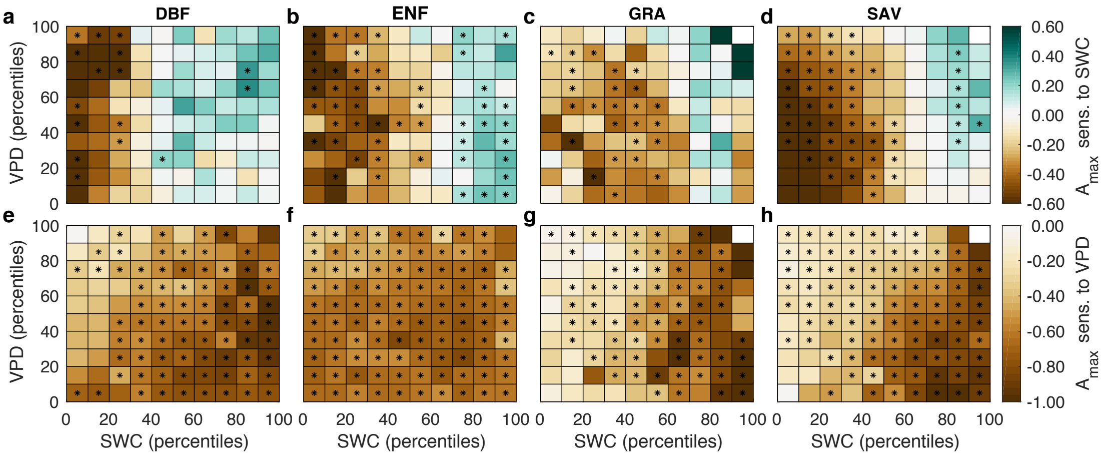

Supplementary Fig. 12 The sensitivity of maximum photosynthetic assimilation rate (A_{max}) to soil water content (SWC) and vapor pressure deficit (VPD) across different plant function types. **a-d**, The sensitivity of A_{max} to SWC across different plant function types. **e-h**, The sensitivity of A_{max} to VPD across different plant function types. DBF: broadleaved deciduous forests; ENF: evergreen needle-leaved forests; GRA: grasslands; SAV: savannas. '*' represents the sensitivities are significantly different from zero by t-tests (p<0.05) across all sites.

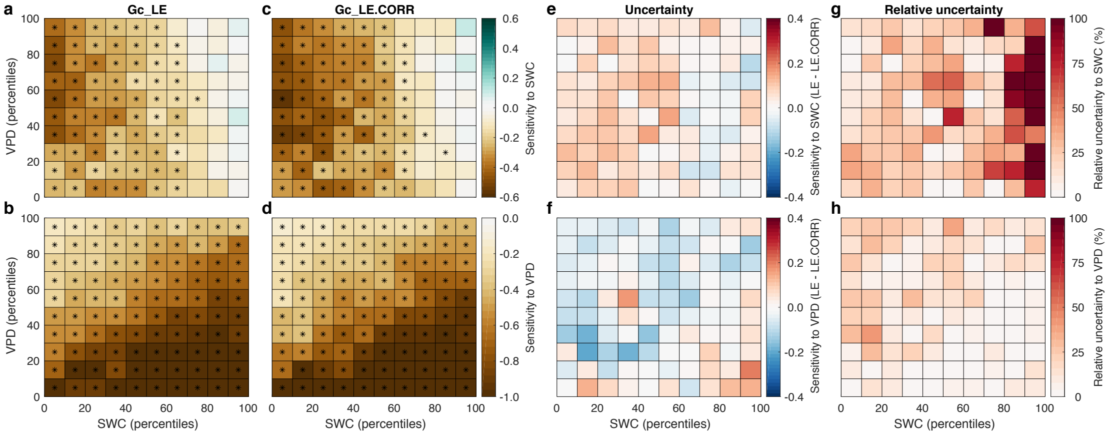

Supplementary Fig. 13 Effect of different latent heat fluxes (LE vs. LE.CORR) on canopy conductance (G_c) sensitivity to soil water content (SWC) and vapor pressure deficit (VPD). **a-b**, The sensitivity of G_c to SWC and VPD using LE (G_c _LE). **c-d**, The sensitivity of G_c to SWC and VPD using LE.CORR (G_c _ LE.CORR). **e-f**, Uncertainty in G_c sensitivity to SWC and VPD. **g-h**, Relative uncertainty in G_c sensitivity to SWC and VPD. Please note that the high levels of relative uncertainty occurred in the bins with statistically insignificant sensitivity values. Since these sensitivity values are close to zero, a low absolute uncertainty leads to a high relative uncertainty.

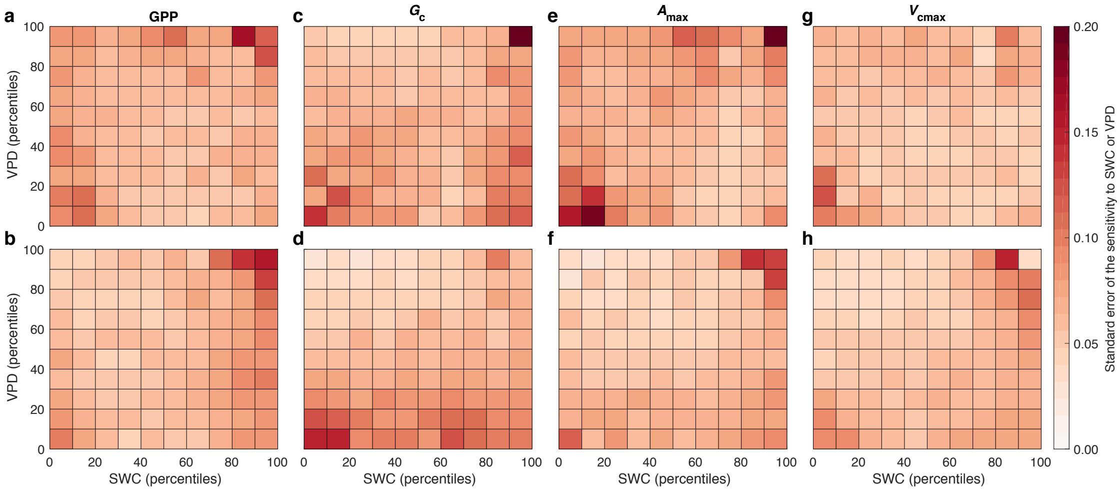

Supplementary Fig. 14 Standard error of the sensitivity of gross primary production (GPP), canopy conductance (G_c), maximum photosynthetic assimilation rate (A_{max}) and maximum carboxylation rate (V_{cmax}) to soil water content (SWC) and vapor pressure deficit (VPD). Standard error of the sensitivity of GPP (**a-b**), G_c (**c-d**), A_{max} (**e-f**), and V_{cmax} (**g-h**) to SWC (**a**, **c**, **e**, **g**) and VPD (**b**, **d**, **f**, **h**) for each bin across all sites.

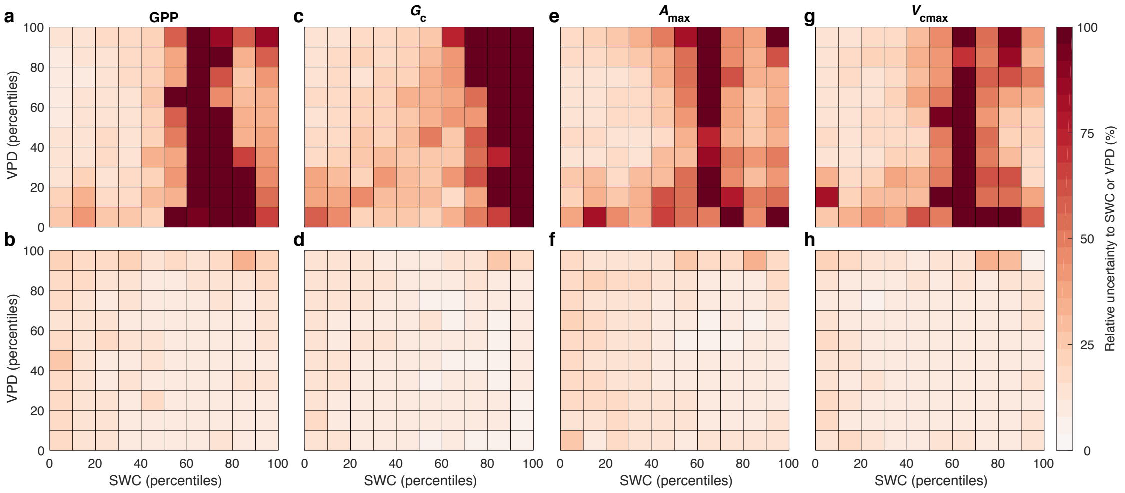

Supplementary Fig. 15 Relative uncertainty of the sensitivity of gross primary production (GPP), canopy conductance (G_c), maximum photosynthetic assimilation rate (A_{max}) and maximum carboxylation rate (V_{cmax}) to soil water content (SWC) and vapor pressure deficit (VPD). Relative uncertainty of the sensitivity of GPP (**a-b**), G_c (**c-d**), A_{max} (**e-f**), and V_{cmax} (**g-h**) to SWC (**a**, **c**, **e**, **g**) and VPD (**b**, **d**, **f**, **h**) for each bin across all sites.

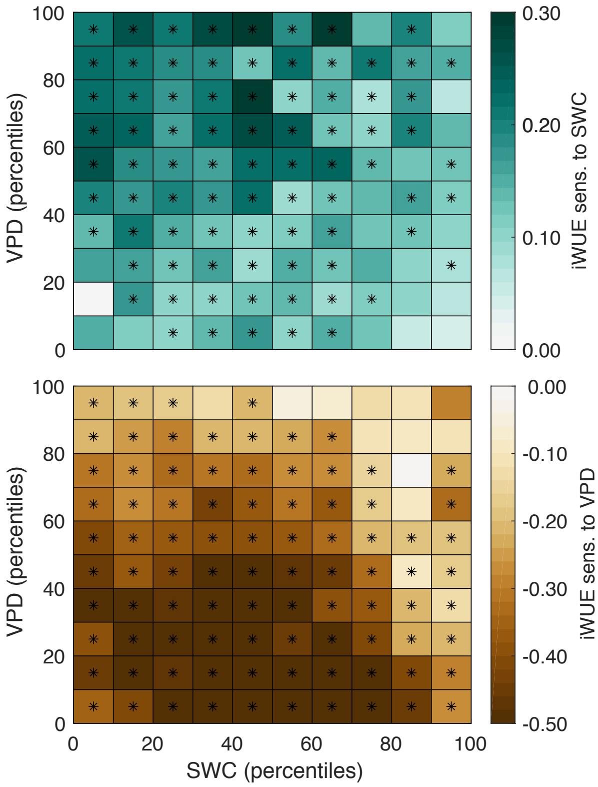

Supplementary Fig. 16 The sensitivity of ecosystem intrinsic water use efficiency (iWUE) to soil water content (SWC) and vapor pressure deficit (VPD) using ICOS and FLUXNET2015 data. Positive signs for the sensitivities indicate that iWUE increases when the SWC becomes drier. Negative signs for the sensitivities indicate that iWUE decreases when VPD decreases. '*' represents the sensitivities are significantly different from zero by t-tests ($p<0.05$) across all sites.

Supplementary Table 1 Information on the 15 flux tower sites with observations during 2014-2018 from the ICOS dataset. Site identifier (ID), latitude (Lat, °), longitude (Long, °), plant functional type (PFT), and study periods are listed.

Site ID	Lat	Long	PFT	Periods	Reference
CH-Cha	47.2102	8.4104	GRA	2014-2018	1
CH-Dav	46.8153	9.8559	ENF	2014-2018	2
CH-Fru	47.1158	8.5378	GRA	2014-2018	1
CH-Lae	47.4781	8.3650	MF	2014-2018	3
CZ-Stn	49.0360	17.9699	DBF	2014-2018	4
DE-Gri	50.9500	13.5126	GRA	2014-2018	5
DE-Hai	51.0792	10.4530	DBF	2014-2018	6
DE-Obe	50.7867	13.7213	ENF	2014-2018	7
DE-Tha	50.9626	13.5652	ENF	2014-2018	8
FR-Hes	48.6741	7.0647	DBF	2014-2018	9
IT-SR2	43.7320	10.2909	ENF	2014-2018	10
IT-Tor	45.8444	7.5781	GRA	2014-2018	11
NL-Loo	52.1666	5.7436	ENF	2014-2018	12
SE-Nor	60.0865	17.4795	ENF	2014-2018	13
SE-Ros	64.1725	19.7380	ENF	2014-2018	13

Supplementary Table 2 Information on the 67 flux tower sites from the ICOS and FLUXNET2015 datasets. Site identifier (ID), latitude (Lat, °), longitude (Long, °), plant functional type (PFT), and study periods are listed. Sites indicated by + were not used for ANNs analysis as described in Methods.

Site ID	Lat	Long	PFT	Periods	Reference
AU-ASM	-22.2830	133.2490	SAV	2010-2013	14
AU-DaP	-14.0633	131.3181	GRA	2007-2013	15
AU-DaS	-14.1593	131.3881	SAV	2008-2014	16
AU-Dry	-15.2588	132.3706	SAV	2008-2014	16
AU-Emr	-23.8587	148.4746	GRA	2011-2013	17
AU-How	-12.4943	131.1523	WSA	2001-2014	18
AU-Rig	-36.6499	145.5759	GRA	2011-2014	19
AU-Stp	-17.1507	133.3502	GRA	2008-2014	16
AU-Tum$^+$	-35.6566	148.1517	EBF	2001-2014	20
AU-Whr	-36.6732	145.0294	EBF	2011-2014	21
AU-Wom	-37.4222	144.0944	EBF	2010-2012	22
AU-Ync	-34.9893	146.2907	GRA	2012-2014	23
BE-Bra$^+$	51.3076	4.5198	MF	2015-2018	24
BE-Vie$^+$	50.3049	5.9981	MF	1996, 1998-2009, 2011-2014, 2016-2018	25
BR-Sa3$^+$	-3.0180	-54.9714	EBF	2000-2003	26
CA-Qfo	49.6925	-74.3421	ENF	2003-2010	27
CH-Cha$^+$	47.2102	8.4104	GRA	2005-2018	1

CH-Fru+	47.1158	8.5378	GRA	2005-2018	1
CH-Lae	47.4781	8.3650	MF	2004-2018	3
CH-Oe1+	47.2858	7.7319	GRA	2002-2008	28
CN-Cng	44.5934	123.5092	GRA	2007-2010	29
CN-Qia	26.7414	115.0581	ENF	2003-2005	30
CZ-BK1	49.5021	18.5369	ENF	2009-2013, 2015-2018	31
CZ-Lnz	48.6816	16.9464	MF	2015-2018	10
CZ-RAJ	49.4437	16.6965	ENF	2016-2018	32
CZ-Stn	49.0360	17.9699	DBF	2010-2018	4
DE-Gri+	50.9500	13.5126	GRA	2005-2018	5
DE-Hai	51.0792	10.4530	DBF	2000-2018	6
DE-HoH	52.0853	11.2192	DBF	2015-2018	13
DE-Obe	50.7867	13.7213	ENF	2008-2018	7
DE-Tha	50.9626	13.5652	ENF	1996-2018	8
DK-Sor	55.4859	11.6446	DBF	1998-2007, 2013-2016, 2018	33
ES-Abr	38.7018	-6.7859	SAV	2015-2018	34
ES-LM1	39.9427	-5.7787	SAV	2015-2018	34
ES-LM2	39.9346	-5.7759	SAV	2015-2018	34
FI-Hyy	61.8474	24.2948	ENF	1998-2017	35
FR-Bil	44.4937	-0.9561	ENF	2016-2018	36
FR-Hes	48.6741	7.0647	DBF	2014-2018	9
FR-LBr	44.7171	-0.7693	ENF	2005-2008	37
GF-Guy	5.2788	-52.9249	EBF	2004-2014	38
IT-CA1	42.3804	12.0266	DBF	2011-2014	39
IT-CA3	42.3800	12.0222	DBF	2011-2014	39
IT-Col	41.8494	13.5881	DBF	1996-2014	40
IT-Cp2	41.7043	12.3573	EBF	2012-2016, 2018	41
IT-Cpz	41.7052	12.3761	EBF	1997-2009	42
IT-Lav	45.9562	11.2813	ENF	2003-2014	43
IT-Lsn	45.7405	12.7503	OSH	2016-2018	13
IT-Noe	40.6061	8.1515	CSH	2004-2014	44
IT-Ro1	42.4081	11.9300	DBF	2000-2008	45
IT-Ro2	42.3903	11.9209	DBF	2002-2012	12
IT-SR2	43.7320	10.2909	ENF	2013-2018	10
IT-SRo	43.7279	10.2844	ENF	1999-2012	46
NL-Loo	52.1666	5.7436	ENF	1997-2018	12
RU-Fyo	56.4615	32.9221	ENF	2000-2013, 2015-2018	47
US-AR1	36.4267	-99.4200	GRA	2009-2012	48
US-Blo	38.8953	-120.6328	ENF	1997-2007	49
US-KS2	28.6086	-80.6715	CSH	2003-2006	50
US-Me2	44.4523	-121.5574	ENF	2002-2014	51

US-Me6	44.3233	-121.6078	ENF	2010-2014	[52]
US-MMS	39.3232	-86.4131	DBF	1999-2014	[53]
US-NR1	40.0329	-105.5464	ENF	1998-2014	[54]
US-SRG	31.7894	-110.8277	GRA	2008-2014	[55]
US-SRM	31.8214	-110.8661	WSA	2004-2014	[56]
US-Ton	38.4316	-120.9660	WSA	2001-2014	[57]
US-Whs	31.7438	-110.0522	OSH	2007-2014	[55]
US-Wkg	31.7365	-109.9419	GRA	2004-2014	[58]
ZA-Kru	-25.0197	31.4969	SAV	2000-2010	[59]

Supplementary Table 3 Five Earth System Models from CMIP6 models with daily outputs used in the analysis. The historical simulation during the 1995-2014 were used.

CMIP6 Models	Institution ID	Modeling Group	Land Component	Land Resolution	Reference
ACCESS-ESM1-5	CSIRO	Commonwealth Scientific and Industrial Research Organisation, Australia	CABLE2.4	1.875° × 1.25°	[60]
CMCC-CM2-SR5	CMCC	Fondazione Centro Euro-Mediterraneo sui Cambiamenti Climatici, Italy	CLM4.5 (BGC mode)	1.25° × 0.94°	[61]
IPSL-CM6A-LR	IPSL	Institute Pierre Simon Laplace, France	ORCHIDEE (v2.0)	2.5° × 1.25°	[62]
NorESM2-LM	NCC	Norwegian Climate Centre, Norway	CLM	2.5° × 1.875°	[63]
NorESM2-MM	NCC	Norwegian Climate Centre, Norway	CLM	1.25° × 0.94°	[63]

Supplementary References

1. Zeeman MJ*, et al.* Management and climate impacts on net CO_2 fluxes and carbon budgets of three grasslands along an elevational gradient in Switzerland. *Agricultural and Forest Meteorology* **150**, 519-530 (2010).
2. Zielis S, Etzold S, Zweifel R, Eugster W, Haeni M, Buchmann N. NEP of a Swiss subalpine forest is significantly driven not only by current but also by previous year's weather. *Biogeosciences* **11**, 1627 (2014).
3. Haeni M*, et al.* Winter respiratory C losses provide explanatory power for net ecosystem productivity. *Journal of Geophysical Research: Biogeosciences* **122**, 243-260 (2017).
4. Krupková L, Havránková K, Krejza J, Sedlák P, Marek MV. Impact of water scarcity on spruce and beech forests. *Journal of Forestry Research* **30**, 899-909 (2019).
5. Hussain M*, et al.* Summer drought influence on CO_2 and water fluxes of extensively managed grassland in Germany. *Agriculture, ecosystems & environment* **141**, 67-76 (2011).
6. Ahrends HE*, et al.* Tree phenology and carbon dioxide fluxes: use of digital photography for process-based interpretation at the ecosystem scale. *Climate Research* **39**, 261-274 (2009).
7. Zimmermann F, Plessow K, Queck R, Bernhofer C, Matschullat J. Atmospheric N-and S-fluxes to a spruce forest—Comparison of inferential modelling and the throughfall method. *Atmospheric Environment* **40**, 4782-4796 (2006).
8. Grünwald T, Bernhofer C. A decade of carbon, water and energy flux measurements of an old spruce forest at the Anchor Station Tharandt. *Tellus B* **59**, 387-396 (2007).
9. Granier A*, et al.* The carbon balance of a young Beech forest. *Funct Ecol* **14**, 312-325 (2000).
10. Gourlez de la Motte L*, et al.* Non-stomatal processes reduce gross primary productivity in temperate forest ecosystems during severe edaphic drought. *Philosophical Transactions of the Royal Society B* **375**, 20190527 (2020).
11. Galvagno M*, et al.* Phenology and carbon dioxide source/sink strength of a subalpine grassland in response to an exceptionally short snow season. *Environmental Research Letters* **8**, 025008 (2013).
12. Gioli B*, et al.* Comparison between tower and aircraft-based eddy covariance fluxes in five European regions. *Agricultural and Forest Meteorology* **127**, 1-16 (2004).
13. Centre DTIET. Drought-2018 ecosystem eddy covariance flux product in FLUXNET-Archive format - release 2019-1. ICOS Carbon Portal. doi:10.18160/PZDK-EF78. (2019).
14. Barraza V*, et al.* Estimation of latent heat flux over savannah vegetation across the North Australian Tropical Transect from multiple sensors and global meteorological data. *Agricultural and Forest Meteorology* **232**, 689-703 (2017).
15. Zhuang W*, et al.* How energy and water availability constrain vegetation water-use along the North Australian Tropical Transect. *International Journal of Plant Production* **10**, (2016).
16. Cernusak LA, Hutley LB, Beringer J, Holtum JA, Turner BL. Photosynthetic physiology of eucalypts along a sub-continental rainfall gradient in northern Australia. *Agricultural and Forest Meteorology* **151**, 1462-1470 (2011).
17. Schroder I, Kuske T, Zegelin S. Eddy Covariance Dataset for Arcturus (2011–2013), Geoscience Australia. Canberra, Tech. rep., doi. org/102.100. 100/14249 (2014).

18. Livesley SJ*, et al.* Seasonal variation and fire effects on CH_4, N_2O and CO_2 exchange in savanna soils of northern Australia. *Agricultural and Forest Meteorology* **151**, 1440-1452 (2011).
19. Azmi M, Rüdiger C, Walker JP. Statistical analysis of short-term water stress conditions at Riggs Creek OzFlux tower site. *Theoretical and Applied Climatology*, 1-13 (2016).
20. Leuning R, Cleugh HA, Zegelin SJ, Hughes D. Carbon and water fluxes over a temperate Eucalyptus forest and a tropical wet/dry savanna in Australia: measurements and comparison with MODIS remote sensing estimates. *Agricultural and Forest Meteorology* **129**, 151-173 (2005).
21. van Gorsel E*, et al.* Carbon uptake and water use in woodlands and forests in southern Australia during an extreme heat wave event in the "Angry Summer" of 2012/2013. *Biogeosciences, 2016, vol 13, núm 21, p 5947-5964*, (2016).
22. Fest BJ, Livesley SJ, von Fischer JC, Arndt SK. Repeated fuel reduction burns have little long-term impact on soil greenhouse gas exchange in a dry sclerophyll eucalypt forest. *Agricultural and Forest Meteorology* **201**, 17-25 (2015).
23. Beringer J, Walker J. FLUXNET2015 AU-Ync Jaxa. FluxNet; Monash University; University of Western Australia https://doi.org/10.18140/flx/1440208 (2016).
24. Gielen B, Verbeeck H, Neirynck J, Sampson DA, Vermeiren F, Janssens IA. Decadal water balance of a temperate Scots pine forest (Pinus sylvestris L.) based on measurements and modelling. *Biogeosciences* **7**, 1247-1261 (2010).
25. Aubinet M, Chermanne B, Vandenhaute M, Longdoz B, Yernaux M, Laitat E. Long term carbon dioxide exchange above a mixed forest in the Belgian Ardennes. *Agr Forest Meteorol* **108**, 293-315 (2001).
26. Miller SD, de Sousa CAD, Menton MC, Maia AR, da Rocha HR, Goulden ML. Effects of selective logging on tropical forest tree growth. *Journal of Geophysical Research: Biogeosciences* **113**, (2008).
27. Giasson MA, Coursolle C, Margolis HA. Ecosystem-level CO_2 fluxes from a boreal cutover in eastern Canada before and after scarification. *Agr Forest Meteorol* **140**, 23-40 (2006).
28. Ammann C, Flechard CR, Leifeld J, Neftel A, Fuhrer J. The carbon budget of newly established temperate grassland depends on management intensity. *Agr Ecosyst Environ* **121**, 5-20 (2007).
29. Dong G. FLUXNET2015 CN-Cng Changling. FluxNet; Shanxi University https://doi.org/10.18140/flx/1440209 (2016).
30. Wang H, Fu X. FLUXNET2015 CN-Qia Qianyanzhou. FluxNet; IGSNRR Chinese Academy of Sciences https://doi. org/10.18140/flx/1440141 (2016).
31. Reichstein M*, et al.* On the separation of net ecosystem exchange into assimilation and ecosystem respiration: review and improved algorithm. *Global Change Biol* **11**, 1424-1439 (2005).
32. McGloin R, Šigut L, Havránková K, Dušek J, Pavelka M, Sedlák P. Energy balance closure at a variety of ecosystems in Central Europe with contrasting topographies. *Agricultural and Forest Meteorology* **248**, 418-431 (2018).
33. Granier A, Pilegaard K, Jensen N. Similar net ecosystem exchange of beech stands located in France and Denmark. *Agricultural and Forest Meteorology* **114**, 75-82 (2002).

34. El-Madany TS*, et al.* Drought and heatwave impacts on semi-arid ecosystems' carbon fluxes along a precipitation gradient. *Philosophical Transactions of the Royal Society B* **375**, 20190519 (2020).
35. Vesala T*, et al.* Effect of thinning on surface fluxes in a boreal forest. *Global Biogeochemical Cycles* **19**, (2005).
36. Deirmendjian L*, et al.* Hydro-ecological controls on dissolved carbon dynamics in groundwater and export to streams in a temperate pine forest. *Biogeosciences*, *15*(2), pp.669-691 (2018).
37. Hibbard K, Law B, Reichstein M, Sulzman J. An analysis of soil respiration across northern hemisphere temperate ecosystems. *Biogeochemistry* **73**, 29-70 (2005).
38. Bonal D*, et al.* Impact of severe dry season on net ecosystem exchange in the Neotropical rainforest of French Guiana. *Global Change Biology* **14**, 1917-1933 (2008).
39. Sabbatini S*, et al.* Greenhouse gas balance of cropland conversion to bioenergy poplar short rotation coppice. *Biogeosciences Discussions* **12**, (2015).
40. Valentini R, Angelis Pd, Matteucci G, Monaco R, Dore S, Mucnozza GS. Seasonal net carbon dioxide exchange of a beech forest with the atmosphere. *Global Change Biology* **2**, 199-207 (1996).
41. Fares S, Savi F, Muller J, Matteucci G, Paoletti E. Simultaneous measurements of above and below canopy ozone fluxes help partitioning ozone deposition between its various sinks in a Mediterranean Oak Forest. *Agricultural and forest meteorology* **198**, 181-191 (2014).
42. Reichstein M*, et al.* Ecosystem respiration in two Mediterranean evergreen Holm Oak forests: drought effects and decomposition dynamics. *Functional Ecology* **16**, 27-39 (2002).
43. Wei S*, et al.* Data-based perfect-deficit approach to understanding climate extremes and forest carbon assimilation capacity. *Environmental Research Letters* **9**, 065002 (2014).
44. Reichstein M*, et al.* Inverse modeling of seasonal drought effects on canopy CO_2/H_2O exchange in three Mediterranean ecosystems. *Journal of Geophysical Research: Atmospheres* **108**, (2003).
45. Rey A, Pegoraro E, Tedeschi V, De Parri I, Jarvis PG, Valentini R. Annual variation in soil respiration and its components in a coppice oak forest in Central Italy. *Global Change Biol* **8**, 851-866 (2002).
46. Chiesi M*, et al.* Modelling carbon budget of Mediterranean forests using ground and remote sensing measurements. *Agricultural and Forest Meteorology* **135**, 22-34 (2005).
47. Moureaux C, Bodson B, Aubinet M. Mesure des flux de CO_2 et bilan carboné de grandes cultures: état de la question et méthodologie. *Biotechnologie, Agronomie, Société et Environnement* **12**, 303-315 (2008).
48. Holmes TR, Hain CR, Anderson MC, Crow WT. Cloud tolerance of remote-sensing technologies to measure land surface temperature. *Hydrology and Earth System Sciences* **20**, 3263 (2016).
49. Goldstein A*, et al.* Forest thinning experiment confirms ozone deposition to forest canopy is dominated by reaction with biogenic VOCs. *Geophysical research letters* **31**, (2004).
50. Powell TL, Bracho R, Li J, Dore S, Hinkle CR, Drake BG. Environmental controls over net ecosystem carbon exchange of scrub oak in central Florida. *Agricultural and Forest Meteorology* **141**, 19-34 (2006).

51. Thomas CK, Law BE, Irvine J, Martin JG, Pettijohn JC, Davis KJ. Seasonal hydrology explains interannual and seasonal variation in carbon and water exchange in a semiarid mature ponderosa pine forest in central Oregon. *Journal of Geophysical Research: Biogeosciences* **114**, (2009).
52. Ruehr NK, Martin JG, Law BE. Effects of water availability on carbon and water exchange in a young ponderosa pine forest: Above-and belowground responses. *Agricultural and forest meteorology* **164**, 136-148 (2012).
53. Schmid HP, Grimmond CSB, Cropley F, Offerle B, Su H-B. Measurements of CO_2 and energy fluxes over a mixed hardwood forest in the mid-western United States. *Agricultural and Forest Meteorology* **103**, 357-374 (2000).
54. Kucharik CJ, Barford CC, El Maayar M, Wofsy SC, Monson RK, Baldocchi DD. A multiyear evaluation of a dynamic global vegetation model at three AmeriFlux forest sites: vegetation structure, phenology, soil temperature, and CO_2 and H_2O vapor exchange. *Ecological Modelling* **196**, 1-31 (2006).
55. Scott RL, Biederman JA, Hamerlynck EP, Barron-Gafford GA. The carbon balance pivot point of southwestern US semiarid ecosystems: Insights from the 21st century drought. *Journal of Geophysical Research: Biogeosciences* **120**, 2612-2624 (2015).
56. Scott RL, Jenerette GD, Potts DL, Huxman TE. Effects of seasonal drought on net carbon dioxide exchange from a woody-plant-encroached semiarid grassland. *Journal of Geophysical Research: Biogeosciences* **114**, (2009).
57. Xu L, Baldocchi DD, Tang J. How soil moisture, rain pulses, and growth alter the response of ecosystem respiration to temperature. *Global Biogeochemical Cycles* **18**, (2004).
58. Scott RL, Hamerlynck EP, Jenerette GD, Moran MS, Barron-Gafford GA. Carbon dioxide exchange in a semidesert grassland through drought-induced vegetation change. *Journal of Geophysical Research: Biogeosciences* **115**, (2010).
59. Williams CA, Hanan N, Scholes RJ, Kutsch W. Complexity in water and carbon dioxide fluxes following rain pulses in an African savanna. *Oecologia* **161**, 469-480 (2009).
60. Ziehn T*, et al.* The Australian Earth System Model: ACCESS-ESM1. 5. *Journal of Southern Hemisphere Earth Systems Science*, (2020).
61. Cherchi A*, et al.* Global Mean Climate and Main Patterns of Variability in the CMCC-CM2 Coupled Model. *Journal of Advances in Modeling Earth Systems* **11**, 185-209 (2019).
62. Lurton T*, et al.* Implementation of the CMIP6 Forcing Data in the IPSL-CM6A-LR Model. *Journal of Advances in Modeling Earth Systems* **12**, e2019MS001940 (2020).
63. Seland Ø*, et al.* The Norwegian Earth System Model, NorESM2–Evaluation of theCMIP6 DECK and historical simulations. *Geoscientific Model Development Discussions*, 1-68 (2020).
